# NestedBD: Bayesian inference of phylogenetic trees from single-cell copy number profiles under a birth-death model

**DOI:** 10.1186/s13015-024-00264-4

**Published:** 2024-04-29

**Authors:** Yushu Liu, Mohammadamin Edrisi, Zhi Yan, Huw A Ogilvie, Luay Nakhleh

**Affiliations:** 1 Department of Computer Science, Rice University, 6100 Main St, Houston, 77005 TX USA; 2 Department of Genetics, University of Texas MD Anderson Cancer Center, TX 77030 Houston, USA

**Keywords:** Copy number aberrations, Single-cell DNA sequencing data, Birth-death model, Phylogenetic inference

## Abstract

Copy number aberrations (CNAs) are ubiquitous in many types of cancer. Inferring CNAs from cancer genomic data could help shed light on the initiation, progression, and potential treatment of cancer. While such data have traditionally been available via “bulk sequencing,” the more recently introduced techniques for single-cell DNA sequencing (scDNAseq) provide the type of data that makes CNA inference possible at the single-cell resolution. We introduce a new birth-death evolutionary model of CNAs and a Bayesian method, NestedBD, for the inference of evolutionary trees (topologies and branch lengths with relative mutation rates) from single-cell data. We evaluated NestedBD’s performance using simulated data sets, benchmarking its accuracy against traditional phylogenetic tools as well as state-of-the-art methods. The results show that NestedBD infers more accurate topologies and branch lengths, and that the birth-death model can improve the accuracy of copy number estimation. And when applied to biological data sets, NestedBD infers plausible evolutionary histories of two colorectal cancer samples. NestedBD is available at https://github.com/Androstane/NestedBD.

**Supplementary Information:**

The online version contains supplementary material available at 10.1186/s13015-024-00264-4.

## Background

Copy number aberrations, or CNAs, are somatic mutations that delete or amplify genomic regions and could cause cancer by amplifying oncogenes [1] or deleting tumor suppressor genes [2]. CNAs are distinguished from copy number variations, or CNVs, which are typically germline mutations that serve as markers for population or evolutionary genetic studies. CNAs can vary in terms of the size of the genomic region that is amplified or deleted, the number of such events across the genome, as well as the rate at which they occur [3]. In particular, a CNA could amplify an entire genome or delete/amplify an entire chromosome [4]. However, CNAs are often smaller, spanning thousands or fewer base pairs [5].

The accumulation of CNAs during cancer development and progression could result in intra-tumor heterogeneity (ITH), where distinct CNA signatures characterize different groups of cells [6]. Elucidating ITH from genomic data is important for the diagnosis, prognosis, and treatment of cancer [2, 6–13]. For example, the adaptive therapy strategy proposed in [14] is designed by taking ITH into account and utilizing it for determining which cells, or clones of cells, to target. Single-cell DNA sequencing (scDNAseq) [15–19] is ideal for inferring CNAs and ITH as it generates DNA sequence data from individual cells that are readily available for comparative genomic and evolutionary analyses [16]. Indeed, several methods have been developed for inferring copy number profiles from scDNAseq data [20], though their accuracy needs improvement [21].

scDNAseq data sets of thousands, and even tens of thousands, of cells will become commonplace, e.g., [22]. Development of “one-step methods” that could infer, with high accuracy, evolutionary histories of such large data sets is likely to prove very challenging. Instead, multi-step methods, where, for example, the data is first clustered, evolutionary histories are then inferred on the individual clusters, and the resulting trees are finally glued together, could prove the approach of choice. This is analogous to the supertree approach to large-scale phylogenetics [23]. Not only is this approach taken for inferring large-scale species phylogenies at the scale of the Tree of Life, but also, for example, for large-scale virus genome data, e.g., [24]. In the case of ITH, one goal would be to infer the tree of clones, which is relatively a small tree, as its leaves correspond to the individual clones, and then infer the trees of individual clones separately. In this study, we focus on this approach and the sub-clonality level, i.e., evolutionary analyses of groups of cells that is assumed to have little or no clonality. In particular, we target the problem of inferring the evolutionary history, along with ancestral copy number of profiles, of a set of individual cells using scDNAseq data, where each cell is defined by its copy number profile. That is, we assume the copy number profiles have been estimated already, and treat them as input (while accounting for error). Furthermore, we focus on focal CNAs that impact sub-chromosomal genomic regions, rather than whole genomes or chromosomes.

SCICoNE [25] and CONET [26] are two recent tools for simultaneous CNA detection and evolutionary history reconstruction on scDNAseq, leveraging the shared evolutionary history among single cells to infer CNAs. In this regard, both SCICoNE and CONET estimate a mutation tree, where a path from the root to a leaf defines the CNA signature of all cells attached to that leaf. In this sense, these two methods do not fit within our study, which, as mentioned above, is focused on sub-clonal inference. We discuss this point further in “Evaluating tree inference on simulated data” section.

In this study, we address the problem of inferring a phylogenetic tree with branch lengths, with the two main goals of our work being to study the appropriateness of (1) an independent-bins assumption in these analyses and (2) a birth-death model of CNAs under this assumption. In studies of CNAs, it is common to partition the genome into bins, where each bin is a fixed number of nucleotides, rather than conduct the analysis at the resolution of individual nucleotides [20]. Given that CNAs naturally span many bins and CNAs could overlap over time, copy numbers in adjacent bins are *not* independent. Trying to model CNAs as events while taking into account such dependencies could result in intractable inference problems. Indeed, the MEDICC model developed by Schwarz et al. [27] aims to capture these dependencies, but inference under this model is very limited in terms of the size of data given the prohibitive computational requirements [28]. While violated in practice, an assumption of independence among sites and loci is commonplace in phylogenetic analyses and method development due to the computational efficiencies it leads to. While models like MEDICC2 [29] address some of the computational limitations, the integration of MEDICC2’s approach, which is focused on computing pairwise distances between CNA profiles, into other methods, remains challenging. Adapting these strategies to other contexts, such as NestedBD, which aims to infer branch lengths and mutation rates without directly addressing bin dependencies, would require significant methodological adjustments. Such complexity suggests that studying the robustness of evolutionary analyses to the independent-bins assumption is still critical due to the appeal and practicality of such an assumption, especially as data sets become larger.

**Fig. 1:**
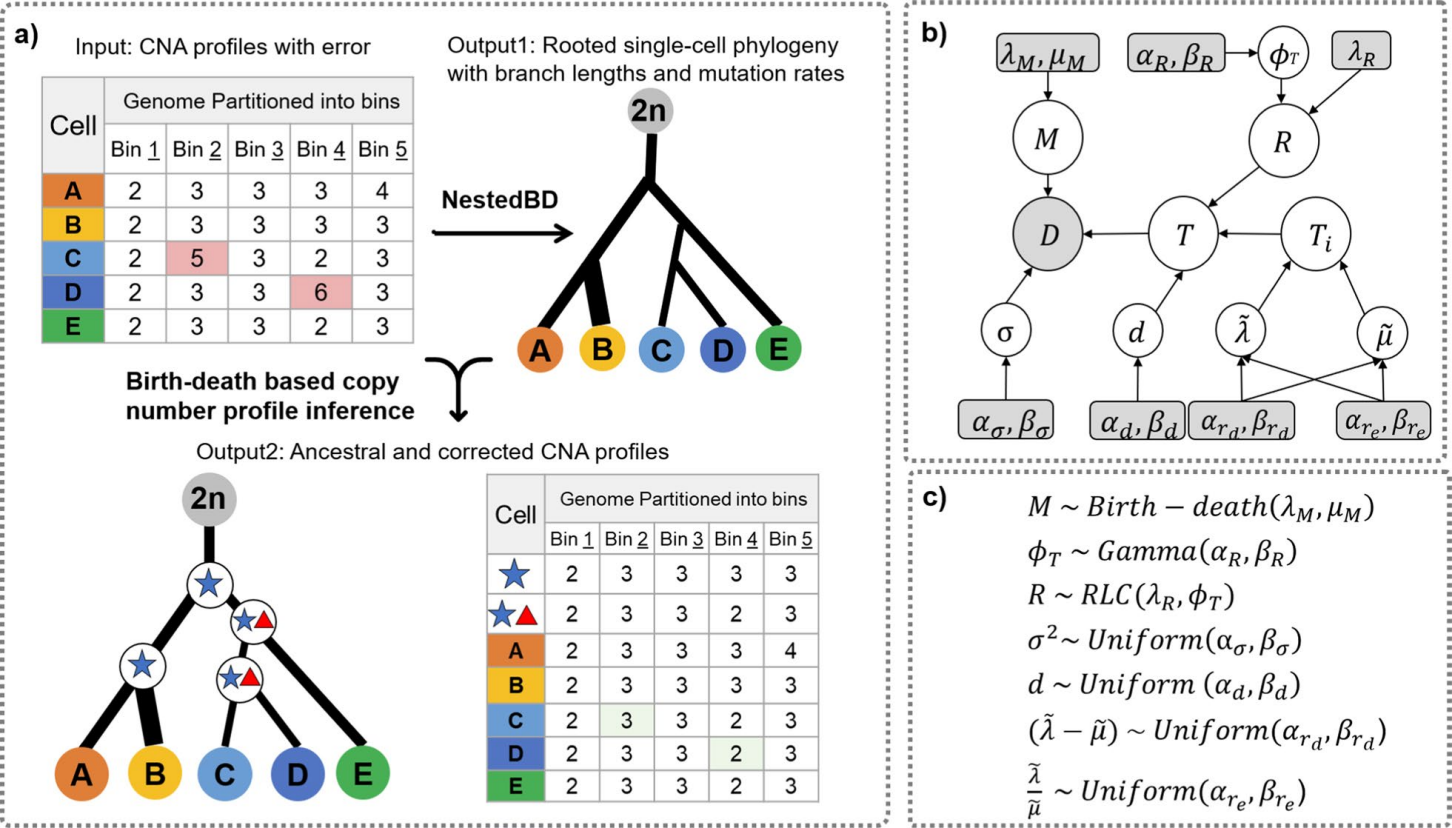
Overview of NestedBD. **a** NestedBD infers a single-cell phylogenetic tree with branch lengths and branch-specific mutation rates from binned copy number profile estimates. Furthermore, the method infers ancestral copy number profiles as well as “corrected” copy number profiles at the leaves. **b** The graphical model underlying NestedBD. Shaded nodes correspond to the observed values or fixed parameters; white nodes are latent variables. **c** Priors and distributions of the variables. The variables are described in detail in “Methods”

Here, we study the impact of assuming that copy numbers across bins are independent of the quality of phylogenetic inference. Furthermore, we propose the first formulation and inference method for copy number profile data from scDNAseq based on a birth-death model of copy numbers. We developed a new method, NestedBD, for Bayesian inference of phylogenetic trees from scDNAseq data under a birth-death model of copy number evolution, assuming the bins are independent. The cells are also assumed to have been sampled at a single time point. NestedBD is implemented as a package in BEAST 2 [30], utilizing existing Markov chain Monte Carlo (MCMC) implementations and allowing for joint inference of trees and model parameters. An overview of NestedBD and its underlying model are shown in Fig. 1.

We assessed the performance of NestedBD on simulated data and compared it to the performance of two commonly used methods, neighbor-joining (NJ) [31] and maximum parsimony (MP) as implemented in PAUP* [32]. These two methods are readily applicable to CNA data since NJ requires pairwise distances among cells, which can be computed from the copy number profiles. MP works directly on the copy number profiles and seeks a tree that minimizes the total number of copy number changes along its branches. Furthermore, these two methods are run in a way that assumes independence among the bins. Additionally, we have included a comparison with Lazac [33], a state-of-the-art method for inferring copy number phylogenies because it has been extensively benchmarked against various methods, demonstrating its effectiveness in copy number phylogeny inference. We found that NestedBD infers more accurate tree topologies than the other methods on the simulated data. In addition, NestedBD can provide accurate estimates of branch lengths in terms of relative times of evolution, which can be scaled by branch-specific mutation rates to obtain an estimated number of copy number changes along each branch if desired. We also explored NestedBD’s applicability on two data sets of colorectal cancer [34] and demonstrated its potential to infer informative evolutionary histories from single-cell data.

## Methods

In this work, we assume that the genomes under consideration are partitioned into bins such that all genomes have the same number and size of bins. The copy number profile of a cell at each bin is an element of $\{0,1,2,\ldots \}$.

### A birth-death evolutionary model of CNAs

To compute the likelihood of phylogeny, we first need an evolutionary model that defines the transition probability between copy number states. We model the copy number amplification and deletion by a constant-rate birth-death process $\{Z(t), t \ge 0\}$ with state space $S = \{0,1,2,....\}$. *Z*(*t*) gives the copy number state of a bin at time *t*. The linear birth-death process first introduced by [35] is also used in [36] to model gene content evolution. We assume each copy is independently amplified with birth rate $\lambda _M > 0$ and deleted with death rate $\mu _M > 0$. The transition rate, which measures the frequency at which the system’s state transitions from one state to another per unit of time, can be computed based on the current copy number state and the birth/death rate. Specifically, at time *t* when the system has a copy number state m, or $Z(t) = m$, the transition rate to state $Z(t+\Delta t) = m+1$ is $m\lambda _M$, corresponding to the occurrence of a birth event. This rate is multiplied by *m* to account for the fact that each of the *m* existing copies has an independent chance to be amplified. Conversely, the transition rate to state $Z(t+\Delta t) = m-1$ is $m\mu _M$, which corresponds to a death event. Similarly, the multiplication by *m* reflects that each of the *m* copies also has an independent chance of being deleted. Note that when $Z(t) = 0$, the transition rate becomes zero, suggesting neither amplification nor deletion from zero is allowed. Then, given the transition rate, we can compute the transition probability between copy number states as follows. Let *i* be the copy number state at the child node, *j* be the copy number state at the parent node, and *t* be the time between the parent node and the child node. Since there is no prior information on the birth and death rates, we assume $\lambda _M = \mu _M = r$. The transitional probability of the birth-death process has been addressed in the works of [37–39], and based on the solutions presented in these sources, we can compute the conditional probability $\textbf{P}(i | j, t)$ as follows:

1 $$\begin{aligned} \textbf{P}(i | j, t) = {\left\{ \begin{array}{ll} 0, &{} \text {if}\ i \ne j = 0 \\ 1, &{} \text {if}\ i = j = 0\\ (\frac{rt}{1 + rt})^j , &{} \text {if}\ i = 0 \ne j\\ \frac{rt^{i-1}}{(1 + rt)^{i+1}} , &{} \!\!\!\!\!\!\!\!\!\!\!\text {if}\ i > 0 \ \& \ j = 1\\ \frac{rt}{rt+1}^{(i+j)} \cdot \sum ^{\min (i, j)}_{k = 1}\left( {\begin{array}{c}i\\ k\end{array}}\right) \left( {\begin{array}{c}j-1\\ k-1\end{array}}\right) (rt)^{-2k},&\text {Otherwise} \end{array}\right. } \end{aligned}$$

### Accounting for error in copy number profile estimates

In practice, copy number profiles are estimated from scDNAseq data and consequently have errors in them [21]. To account for errors in the inferred profiles, we assume that the estimated copy numbers follow a normal distribution centered at the true copy numbers with a constant variance. Specifically, for estimated copy number $c_i$ and true copy number $c_t$, we have $c_i \sim \mathcal {N}(c_t, \sigma ^2)$, which leads to $P(c_{i} |c_{t}, \sigma ) = \frac{e^{-\frac{1}{2}(\frac{c_i - c_t}{\sigma })^2}}{\sigma \sqrt{2\pi }}$.

### Bayesian inference

Given the birth-death evolutionary model of copy number profiles, we use MCMC to sample from the following posterior distribution:

$$\begin{aligned} \textbf{P}(\mathcal {T}, d, \theta , \sigma | D) \propto f(D|\mathcal {T}, d,\sigma ) \textbf{P}(\mathcal {T} |\theta , R)f(\theta )f(d) f(R|\lambda _{R}, \alpha _R, \beta _R), \end{aligned}$$

where *D* is the estimated copy number profile, $\sigma$ is the variance of error in the estimated copy number profile, $\theta$ is the collection of parameters that define a birth-death tree prior on $\mathcal {T}$, (Note that there are two birth-death processes employed in this work—one on the shape of the trees and another on the copy number states. They are distinct and should not be confused.) and *d* is the distance between the common ancestor of all cells and its diploid ancestor. *R* corresponds to the clock model on mutation rates among branches, parameterized by $\lambda _R$, the expected number of rate changes along the tree branches, and the pair $(\alpha _R, \beta _R)$, which defines a gamma distribution on the rate multiplier. We employ standard tree moves as available in BEAST 2 [30] to explore the tree space. Details of the tree moves used in NestedBD are available in Additional file 1.

#### Prior

 We assume the topology $\mathcal {T}$ follows a two-parameter birth-death prior. Specifically, the birth-death model on the tree is a continuous-time process with two parameters, $\tilde{\lambda }$ and $\tilde{\mu }$, the instantaneous per-lineage rates of speciation and extinction. Both $\tilde{\lambda }$ and $\tilde{\mu }$ are constant across the tree in their original characterizations [40]. For the purpose of inference, we parametrize the model using the diversification rate $r_d = (\tilde{\lambda } - \tilde{\mu })$ and extinction fraction $r_e = (\tilde{\mu } / \tilde{\lambda })$. Since there is no prior information on the diversification rate and extinction fraction, we assume a uniform prior on both $r_d$ and $r_e$ with $r_d \sim Uniform(0, 1000000)$ and $r_e \sim Uniform(0, 1)$. Mutation rates on branches are assumed to follow the random local clock (RLC) model, in which each branch either inherits its parent branch rate or, with a certain probability, assumes a new rate drawn from a shared rate distribution [41]. We assume a Poisson prior on the number of rate changes with an expected value $\lambda _{R} = \log {2}$. This sets a 0.5 prior probability on the hypothesis of no change in mutation rate across the phylogeny. We also assume that rate multipliers $\phi _T$ are independently gamma distributed with $\alpha _R = 0.5$ and $\beta _R = 2$ as in [41]. We use the minimum (0) and maximum (9) copy number states considered in this study to set the lower and upper bounds on the variance of error $\sigma ^2$ in the estimated copy number state, which is assumed to be uniformly distributed. Specifically, we set $\alpha _\sigma = 0$ and $\beta _\sigma = 9$.

#### Likelihood

Assuming $r=1$ with transition probability defined by birth-death evolutionary model on copy number state in Eq. (1), we used a modified Felsenstein’s algorithm [42] to compute the likelihood of tree $\mathcal {T}$ constructed from input copy number profile data *D*. We assume a diploid common ancestor of all tumor cells.

We define the state space of copy numbers as $S = \{0, 1, 2,...k\}$, where $k \in \mathbb {N}$ defines the maximum copy number state to be considered during likelihood computation. For flexibility of the method, *k* is left to be a user-specified input with default being 9 considering the maximum value commonly observed in copy number state of cancerous cells. Note that although the likelihood computed under a larger *k* could be more accurate, it may not always be desirable computationally given the likelihood computation takes $\mathcal {O}(nk^2)$ time, where *n* is the number of leaves in the tree.

To compute the likelihood of a tree, we adopt Felsenstein’s algorithm with slight modification when computing the likelihood at the root to account for the diploid origin. Specifically, the original algorithm computes likelihood across the whole tree using conditional likelihoods for all possible states at the root of the tree by $\mathcal {L} = \sum _{x \in S} \pi _x \cdot \mathcal {L}_{\text {root}}(x)$, where *x* refers to the copy number state at the root node of the tree and $\pi _x$ refers to the corresponding prior probability of that copy number state at the root of the tree, and $\mathcal {L}_{\text {root}}(x)$ refers to the conditional likelihoods for the state *x* for the sub-tree under the root (which is the entire tree). Our algorithm computes instead $\mathcal {L} = \sum _{{x \in S}}\textbf{P}(x |2, d) \cdot \mathcal {L}_{\text {root}}(x)$, where $\textbf{P}(x |2, d)$ corresponds to the transition probability as defined in Eq. (1) and *d* represents the time between the diploid and common ancestor of all cancerous cells. By default, *d* is inferred jointly with the topology by assuming a uniform prior $d \sim Uniform(0.001, 5)$ on it.

### Inferring corrected copy number profile

For the inference of corrected copy number profile with an input tree using the birth-death evolutionary model, we applied a dynamic programming algorithm adopted from [43] which maximizes the joint likelihood given the binned copy number profiles of the single cells and tree topology. The transition probability was computed by Eq. (1). To account for the diploid origin of all cells, we computed the probability of each state at the root by $\textbf{P}(x |2, d)$ as defined in “Likelihood” section. While the algorithm was designed for ancestral profile reconstruction while keeping the profiles at the leaves unmodified, it could be paired with an error model to enable correction of the profile estimates at the leaves as follows (for an arbitrary bin). For each leaf *y* of the tree and for each possible copy number *j*, we compute $L_y(j|c_y,E)$, the likelihood of copy number state at *y* being *j* given the estimated copy number state $c_y$ and the error model *E* in “Accounting for error in copy number profile estimates” section. Then given the father of *y* is assigned state *i*, we compute the likelihood of the best reconstruction of leaf *y* by $\max _j L_y(j|c_y,E)P_{ij}(t_y)$, and set the copy number at leave *y* by $C_y = \textrm{argmax}_j L_y(j|c_y,E)P_{ij}(t_y)$, where $P_{ij}$ is the transition probability computed by Eq. (1) and $t_y$ is the length of the branch between *y* and its parent.

### Evaluating tree inference on simulated data

To assess the performance of NestedBD under various scenarios, we designed a simulation study that varies the number of single cells sampled (the leaves in the trees) and the number of CNAs.

#### Simulation protocol

To simulate data with a known ground truth tree, we made a few modifications to the CNA evolutionary simulator described in [21]. First, we randomly sample the allele on which the CNA is going to occur from the paternal and maternal alleles with a binomial distribution with $p=0.5$. Then, we linearize the genome and sample the genomic coordinate *x* where the CNA occurs from a probability distribution whose density function is given by $f(x) \propto \exp (\sum _{i = 1}^{30}\sin (1000x + \phi _i) \cdot \lambda _i)$. We fixed $\phi _i \sim Uniform( -\pi , \pi )$ and $\lambda _i \sim Uniform (0, a)$, where *a* is a user-specified parameter that controls the non-uniformity of the distribution. Note that when setting $a = 0$, the position of the CNA is sampled from a uniform distribution as in the original simulator. Using a normalized sum of sines with a random phase as the distribution to sample copy number events provides enough randomness and allows control of overlap during simulation. For the purpose of this study, we set $a = 0.6$ as we found the CNAs simulated under such setting best resembled what we observed from the biological data sets. On average, more than 90% of the CNAs overlapped with at least one other CNA. Also, the simulator now accepts a user-defined tree and simulates CNAs along the branches of the input tree. In order to simulate CNAs that resemble the patterns of those seen in real data, we took the two trees reconstructed by NestedBD on the colorectal cancer data, which contain 20 and 50 cells, as our model trees (see “Analysis of colorectal cancer samples” section). Specifically, the number of CNAs on a branch with length *t* is sampled from a Poisson distribution with the mean $c \cdot t$, where *c* is the event multiplier that controls the number of CNAs at the leaves of the tree. In order to evaluate how the complexity of copy number events can affect the performance of each method, we set *c* to 90, 125, and 250, corresponding to the cases of low, medium, and high frequency of copy number events, respectively. An example of copy number profiles simulated with different values of *c* is available in Additional file 1: Fig. S2. The size of each CNA event is determined by sampling from an exponential distribution with mean = 10 Mbp, plus a minimum CNA size of 2 Mbp. Whether the CNA resulted in a gain or a loss of copy is determined by sampling from a binomial distribution with $p = 0.5$. After all CNA events are added along the branches, the genome is divided into 15k non-overlapping bins, and the copy number of each bin in a leaf is calculated from all copy number events along the path from the root to the leaf.

Finally, to obtain copy number profile estimates, we aligned the reads generated by the simulator back to the reference genome using BWA [44] with default settings and inferred the profiles at the leaves using Ginkgo [45], as it has been shown to be among the most accurate approaches for estimating copy number profiles [21]. The estimated copy number profiles had a median error rate of 0.24 when compared to true copy number profiles available from the simulator. The distribution of copy number profile estimation error is given in Additional file 1: Fig. S1.

#### Inference methods

We ran NestedBD, MP, NJ, and Lazac on estimated copy number profiles estimated by Ginkgo. To satisfy the independent-bins assumption, we sampled the bins with a 1/20 sampling rate before making the copy number profile data available to all inference methods.

For each simulated data set, NestedBD was run using the BEAST 2 implementation with coupled-MCMC [46], where one chain is “cold,” operating like a standard MCMC, while the other chains are “heated,” making larger state space jumps and proposing new states for other chains, to enhance exploration efficiency. Five chains with random seeds were run for 80 million iterations to assess the convergence of MCMC. The first 20% of posterior samples were discarded as burn-in. To summarize the posterior distribution, 2000 samples were taken from the MCMC chain for computation of inferred topology and branch lengths. We computed the maximum clade credibility tree (MCC; the tree with the maximum product of the posterior clade probabilities) with branch lengths summarized using the median node heights across samples as the point estimates. Specifically, for every possible clade *i* (a group consisting of a single ancestor and all its descendants), we first calculated the posterior probability $p_i$, defined as the proportion of trees in which that clade appears. Then, for every tree *T* in the samples, we compute the product of the posterior probabilities $\prod _i p_i(T)$, where $p_i(T)$ is the posterior probability of clade *i* in tree *T*, and select the tree with maximum product as the MCC tree, $T_{MCC}$, i.e., $T_{MCC} = \mathop {\mathrm {arg\,max}}\limits _T\prod _i p_i(T)$. Branch lengths are summarized by taking the median of the node heights across all trees in which the clade appears, which provides a central estimate of time or evolutionary change for each node. We obtained 100 bootstrap replicates for each data set using both MP and NJ with PAUP* [32] and Lazac with random sampling of bins. Note that MP, NJ and Lazac return unrooted trees by default while NestedBD infers a rooted tree by assuming a diploid origin. To root the trees inferred by MP, NJ and Lazac, we added to each data set a diploid “genome” as an outgroup. For MP, we define the character set as all integers, treating a change of gaining/losing a single copy in any bin as a single mutation. In the case of NJ, we compute the pairwise distance using Hamming distance. Further detailed parameters and options used when running MP and NJ are available in Additional file 1.

As we mentioned in “Background” section, there are methods, such as SCICoNE [25] and CONET [26], that assume clonal evolutionary histories. As such, they infer trees whose leaves correspond to clones and internal nodes correspond to events where the path from the root to a leaf describes the set of events (CNAs) that define the clone at that specific leaf. All cells that share the same CNAs of a given clone are then attached to the clone’s corresponding leaf. Therefore, when cells are analyzed at the sub-clonal level (i.e., cells coming from a single clone), these methods could lump all cells together on a tree with a single node. This is not a limitation of these methods; instead, they are designed for application to data sets where clonality exists. Indeed, this is what we observed when we ran SCICoNE [25] and CONET [26]. As discussed above, we used copy number estimates from read counts of aligned reads generated by the simulator as input to NestedBD, whereas in the studies reporting on SCICoNE and CONET, read counts are directly simulated from true copy number profiles. To make the results comparable to those of NestedBD, we first attempted to run both SCICoNE and CONET at the single-cell level on one of our simulated data sets with 50 cells using corrected read counts from aligned reads. SCICoNE resulted in a mutation tree with a single node, to which all CNA events are assigned, as shown in Additional file 1: Fig. S3, and CONET failed to initialize a tree with a properly defined likelihood. We then ran SCICoNE and CONET on read counts simulated from true copy number profiles, as available from our simulator, using the simulation model of each method. We observed that SCICoNE and CONET generated a tree with 2 and 3 event nodes, respectively.

NestedBD, on the other hand, does not assume clonality. In fact, we envision NestedBD to be run on clonal genotype data that are obtained as representative of clonal copy number profiles or on the sequence data obtained from a single clone. During the tree search process, NestedBD searches for a full binary tree, that is effectively the mutation tree on the ancestors of clones or the tree of cells within one clone. An example of such analysis is the study of [47], where evolutionary histories of renal cancers are estimated, and heterogeneity is explored within the primary tumor and its metastatic sub-clones. Another notable example is the study of [48], in which the authors concentrated on reconstructing subclonal lineage dynamics by inferring multiple trees to delineate the phylogenetic relationships at the single-cell level within the clonal population. As clonality is no longer dominant under this scenario, methods relying on such assumptions would be less applicable. To summarize, methods such as SCICoNE and CONET are designed for different purposes and scenarios like the ones assumed in our study, hence their exclusion from our analysis and, instead, including methods such as maximum parsimony, neighbor-joining, and Lazac.

#### Tree scaling

Branch lengths inferred by NestedBD are not in the same unit as those in the true tree. Therefore, to assess the accuracy of branch length reconstruction, we needed to scale the inferred phylogeny before comparing branch lengths. Given an inferred tree $\mathcal {T}$ and a true tree *R* with same set of leaves, we found the scale factor by computing an OLS regression as follows. Let the set of clades in $\mathcal {T}$ be $\mathcal {T}_C$ and the set of clades in *R* be $R_C$.

We compute $\beta$ that minimizes the residue, $R(\beta ) = ||{\textbf{Y}} - \beta {\textbf{X}}||^2$, where ${\textbf{Y}}$ is a vector of true node heights and ${\textbf{X}}$ is a vector of inferred node heights of clades in $\mathcal {T}_C \cap R_C$. To compute the 95% highest posterior density (HPD) intervals and $R^2$ measures from posterior samples of NestedBD, we summarized the trees from the posterior distribution by MCC trees with median node heights. We then computed the scaling factor of the MCC tree with respect to the true tree for each simulated data set. The same scaling factor was used to scale all selected samples from the posterior distribution.

#### Evaluating accuracy of copy number estimates on simulated data

We used the same simulated data to evaluate the performance of copy number estimation. Hamming distance and L1 norm between true and inferred profiles were used for measuring accuracy.

## Results

### Performance on simulated data

#### Accuracy of inferred topologies

**Fig. 2:**
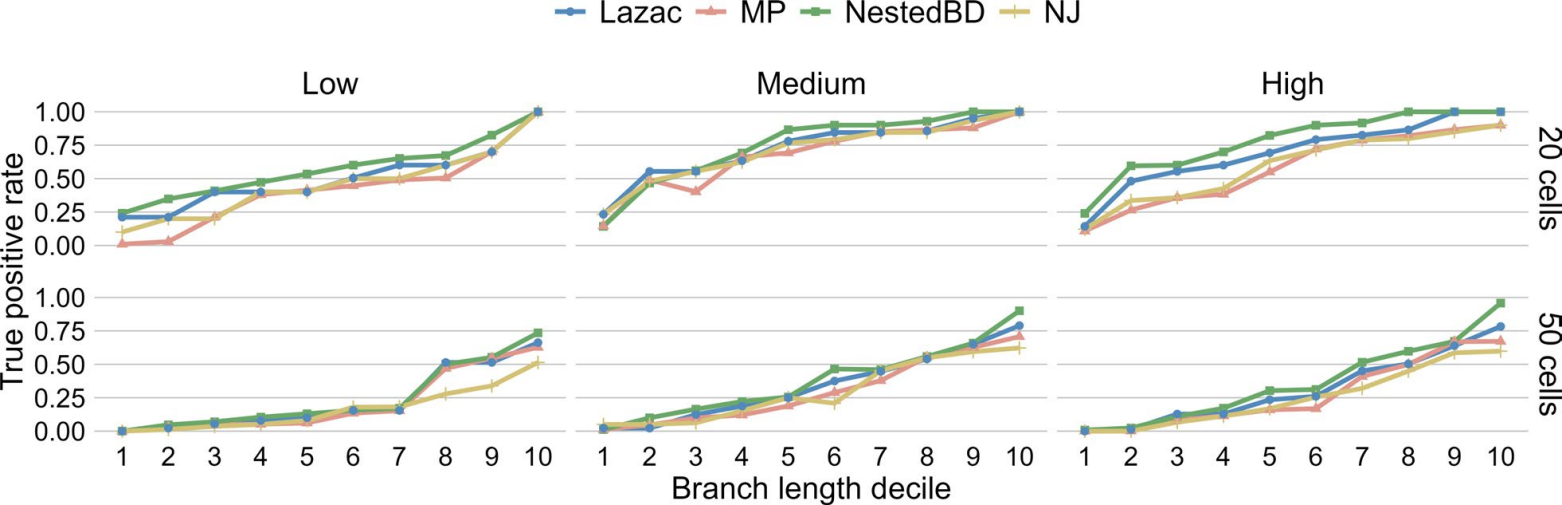
Accuracy of the inferred trees on the simulated data. True positive rates of branches in the true tree vs. lengths of the branches, grouped into deciles, for trees inferred from data sets with 20 cells (top) and 50 cells (bottom). Each point corresponds to a branch length decile in the true tree and the proportion of trees (in the posterior or bootstrap samples) that have that branch, inferred by each of the three methods. ‘Low,’ ‘Medium,’ and ‘High’ correspond to the extent of CNAs in the simulated data sets (see main text)

While it is common to calculate the Robinson-Foulds (RF) distance [49] between the inferred tree and true tree to quantify their difference, this is not particularly useful in our case as there are several groups of cells where cells within each group are equidistant from each other, and their resolution in a binary tree is arbitrary. The RF distance would heavily penalize resolutions that differ from the true one. Therefore, we evaluated a method’s accuracy in inferring branches of the true tree according to their lengths. Specifically, for each branch length, we calculated the true positive rate by counting the number of branches of that length that were correctly inferred by the method. We then grouped branches into deciles according to the branch length and summarized the true positive rate for every decile to study how the accuracy of each method changes with increasing branch lengths. The results are shown in Fig. 2.

As expected, the longer a branch in the true tree, the more likely it is to be recovered. The accuracy of all methods is lower for larger trees. Among all methods, NestedBD has the best accuracy under all event settings, regardless of the number of cells. We also observed that MP and NJ achieved their best accuracy at the medium number of event cases, while the accuracy of NestedBD is less impacted by the extent of CNAs in the data set. A possible explanation of this observation is that for small numbers of CNAs (Low), there is weak signal for MP and NJ to recover the tree, and for a large number of events (High), the overlap of CNAs resulting in more challenging patterns for these two methods. While such trend is less applicable to Lazac, potentially due to the ability of the proposed ZCNT distance to account for bin dependencies [33], NestedBD maintains the highest accuracy under most complex scenarios.

#### Accuracy of estimated branch lengths

To the best of our knowledge, NestedBD is the first method that utilizes a probabilistic birth-death model of CNAs to infer the branch lengths that represent the relative evolutionary time between nodes. In the context of cancer, the branch length translates to the time between evolutionary events or the accumulation of mutations, reflecting the timing of division between the cancer lineages when reconstructing the evolutionary history of cancer cells using copy number profiles. For data with tumor cells sampled with temporal information, the branch length can be mapped to real time, with the potential to guide cancer treatment decisions by showing the timing of key mutations and changes. NestedBD also infers branch-specific mutation rates jointly with the tree topology to provide information on the number of mutations that occurred along each branch. Details of the models we used for branch lengths and mutation rates are available in “A birth-death evolutionary model of CNAs” section. The number of mutations estimated by MP could be used to obtain an estimate of branch lengths. Similarly, the pairwise distances utilized by NJ to infer the evolutionary tree could be used as proxies for branch lengths. We assessed the accuracy of branch lengths by focusing on (1) the accuracy of the estimated branch lengths in terms of the relative time of evolution, which is only available from NestedBD, and (2) the accuracy of the estimated branch lengths in terms of the number of mutations, which is available for all three methods.

**Fig. 3:**
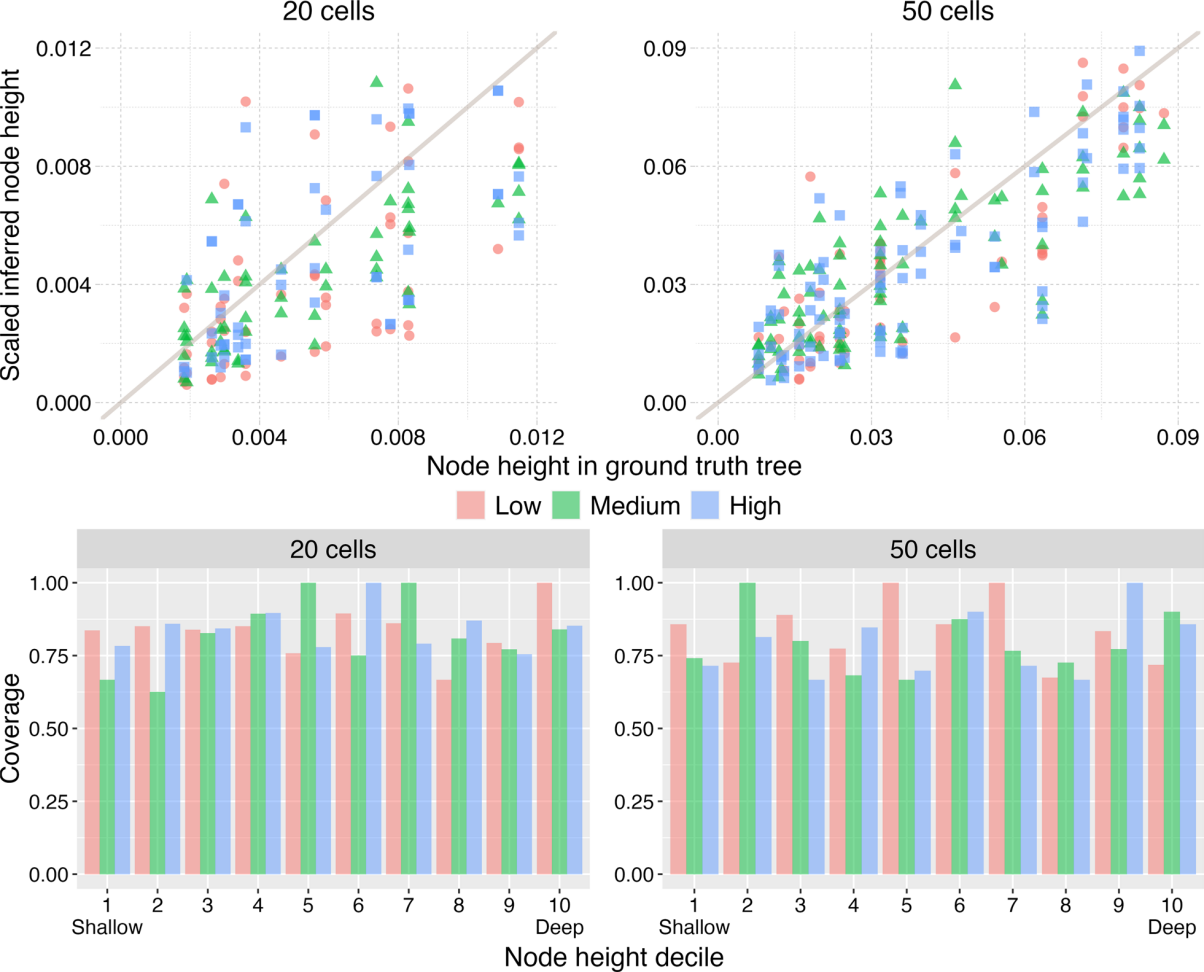
Node heights estimated by NestedBD on the simulated data. (Top) The scaled inferred node heights vs. true node heights from trees inferred by NestedBD from simulated data sets with 20 cells (left) and 50 cells (right). (Bottom) Percentage of true node heights that lies in the 95% HPD interval inferred by NestedBD from simulated data sets with 20 cells (left) and 50 cells (right). ‘Low,’ ‘Medium,’ and ‘High’ correspond to the extent of CNAs in the simulated data sets (see main text)

For NestedBD, we summarized the accuracy of each inferred branch length—which represents the division time of a lineage—by calculating the coverage of the posterior distribution of the node heights. Details of how the posterior distribution is defined are available in “Bayesian inference” section. Since the inferred and true tree topologies could differ, we only compared the heights of the nodes in the true tree that had corresponding nodes in the inferred tree (two nodes are corresponding if and only if the sets of leaves under them are identical). Furthermore, as described above, the node heights were scaled to ensure comparability between the true and inferred node heights. Figure 3 summarizes the node heights inferred by NestedBD for all the simulated data sets after scaling (described in “Tree scaling” section).

For a large number of nodes, the median of inferred node heights in the posterior samples appears to be a reasonable point estimate of the true node height, and the true node height is within the predicted 95% highest posterior density (HPD) interval (see “Tree scaling” section for description of HPD).

We summarized the accuracy of the branch lengths in terms of the number of mutations using the pairwise distances between the leaves in the inferred topology. The pairwise distance between two leaves was calculated by counting the number of mutations on the branches on the unique simple path between the two leaves on the phylogenetic tree. As discussed above, MP and NJ infer the branch lengths corresponding to the number of copy number changes over the genome length, and therefore, no preprocessing of their results was required for our evaluation. For NestedBD, the inferred branch lengths were scaled by the branch-specific mutation rate to provide information on number of mutations on the target branch.

**Fig. 4:**
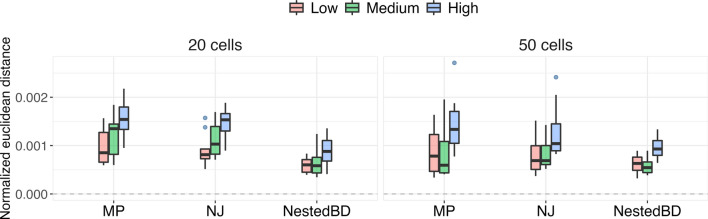
Accuracy of estimated branch lengths in units of number of copy number changes on the simulated data. The box plots show the distributions of normalized Euclidean distances between true and inferred pairwise distances between leaves in terms of number of mutations. Methods are MP, NJ, and NestedBD. ‘Low,’ ‘Medium,’ and ‘High’ correspond to the extent of CNAs in the simulated data sets (see main text). The pairwise distances are summarized based on 10 replicates

Similar to the evaluation of node heights, the pairwise distances between the leaves in both ground truth trees and inferred trees were normalized before comparison. Specifically, given an inferred tree *T* and a true tree *R* with the same set of leaves, *L*, we first calculated the pairwise distance $d_{ij}$ for all pairs of leaves $i, j \in L$ ($i \ne j$). For each method, we then performed the min-max normalization on the pairwise distance matrix *D* using the formula $n_{ij} = \frac{d_{ij} - \min (D)}{\max (D) - \min (D)}$. We then computed the Euclidean distance between $N_T$ and $N_R$ by $\frac{||N_T - N_R||_2}{(|L|-1)^2}$ for each replicate where $N_{R}$ and $N_{T}$ are the normalized pairwise distance matrices of the true tree and inferred tree, respectively. The results are summarized in Fig. 4. As the figure shows, NestedBD obtains more accurate estimates of branch lengths in terms of the number of mutations compared to MP and NJ while providing the timing of lineage division and mutation rate on the specific branch.

#### Accuracy of copy number estimation

**Fig. 5:**
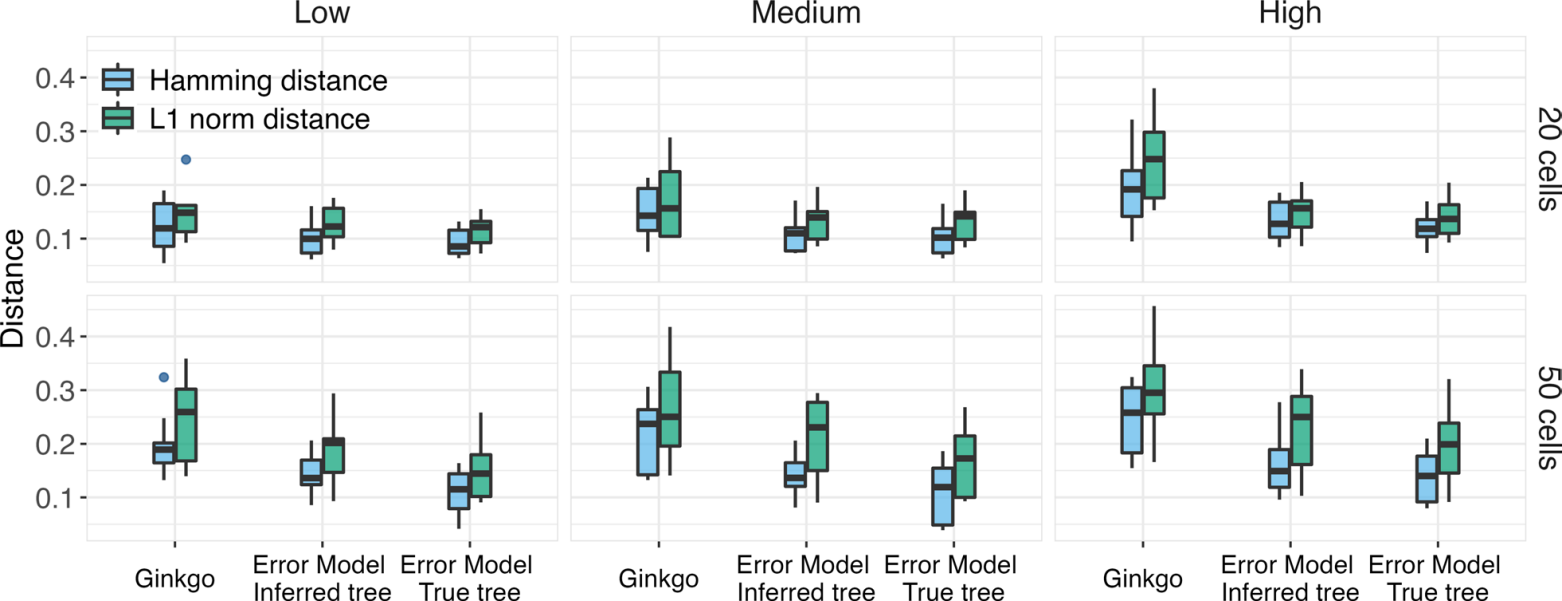
Accuracy of copy number correction. The box plots show the distributions of copy number profile estimation error at the leaves of the trees measured by the Hamming distance and L1 norm between the true and inferred copy number profile. The three inference methods used are Ginkgo, our proposed birth-death-based method with error modeling using the inferred and true tree. ‘Low,’ ‘Medium,’ and ‘High’ correspond to the extent of CNAs in the simulated data sets (see main text)

We proposed in “Inferring corrected copy number profile” section an algorithm that simultaneously corrects copy number profiles at the leaves of a given tree, as well as infers ancestral copy number profiles at the internal nodes of the tree. To assess the performance of the algorithm, for each simulated data set, we inferred the copy number with both the true tree and the maximum clade credibility (MCC) tree inferred by NestedBD (see “Inference methods” section for details of the MCC tree). The former allows us to assess the accuracy of methods assuming the tree is correct, whereas the latter allows us to factor in the tree estimation error when computing the accuracy of the estimated copy number. The results of correcting the copy number profiles at the leaves are shown in Fig. 5.

As the results show, our proposed birth-death-based method with error modeling improves the copy number profile estimated by Ginkgo [45]. Naturally, the results are better when the true (correct) tree is used, but even when the estimated tree is used, the method improves upon Ginkgo. Furthermore, while the extent of CNAs has an impact on the accuracy of copy number estimation for all methods, Ginkgo is more affected by the increasing number of copy number events. At low event settings, the accuracy of Ginkgo is still comparable to that of our proposed method using inferred/true tree. The advantage of our proposed method becomes more significant under medium and high event settings, possibly due to the ability of our birth-death process to handle recurrent mutations at the same locus.

**Fig. 6:**
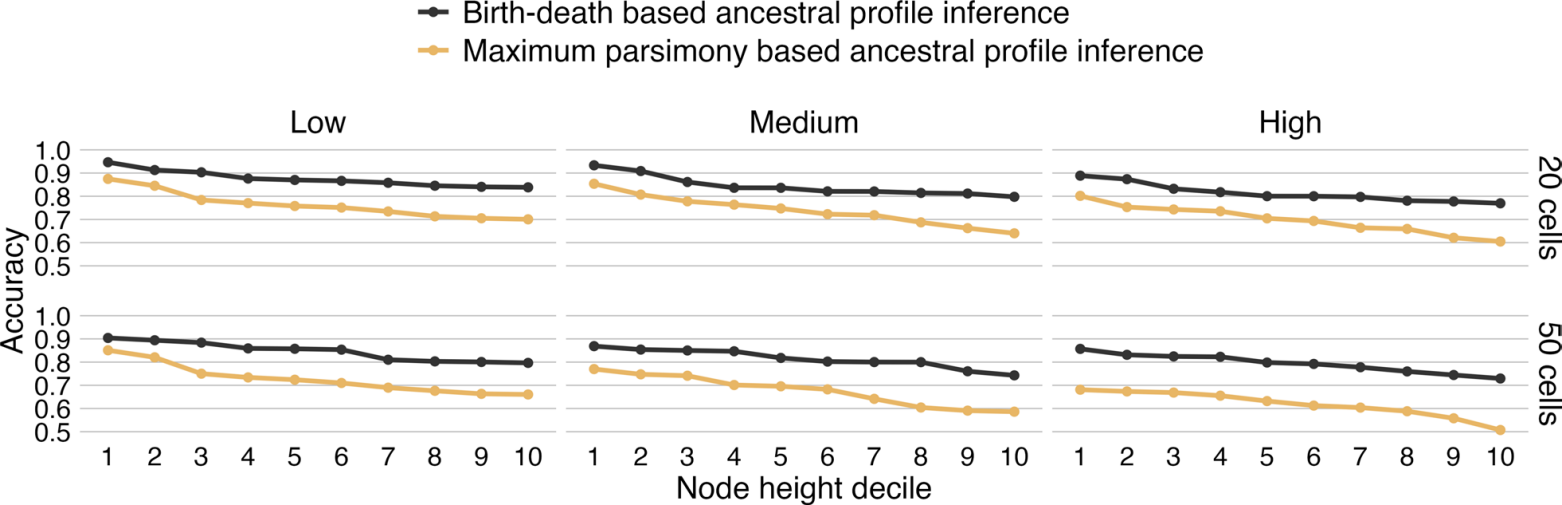
Accuracy of ancestral profile inference. The accuracy of inferred ancestral profiles as a function of the heights of their corresponding internal nodes, grouped into deciles, for data sets with 20 cells (top) and 50 cells (bottom). Ancestral inference was performed using our proposed birth-death-based method with error modeling as well as maximum parsimony. In both cases, the true tree was assumed. ‘Low,’ ‘Medium,’ and ‘High’ correspond to the extent of CNAs in the simulated data sets (see main text)

We also assessed the accuracy of ancestral profiles reconstructed by our birth-death-based method and compared it to that obtained from MP given the true tree, as only in such a case there is a one-to-one correspondence between the internal nodes. We observed that our proposed method achieves higher accuracy under all scenarios evaluated, as shown in Fig. 6.

### Analysis of colorectal cancer samples

**Fig. 7:**
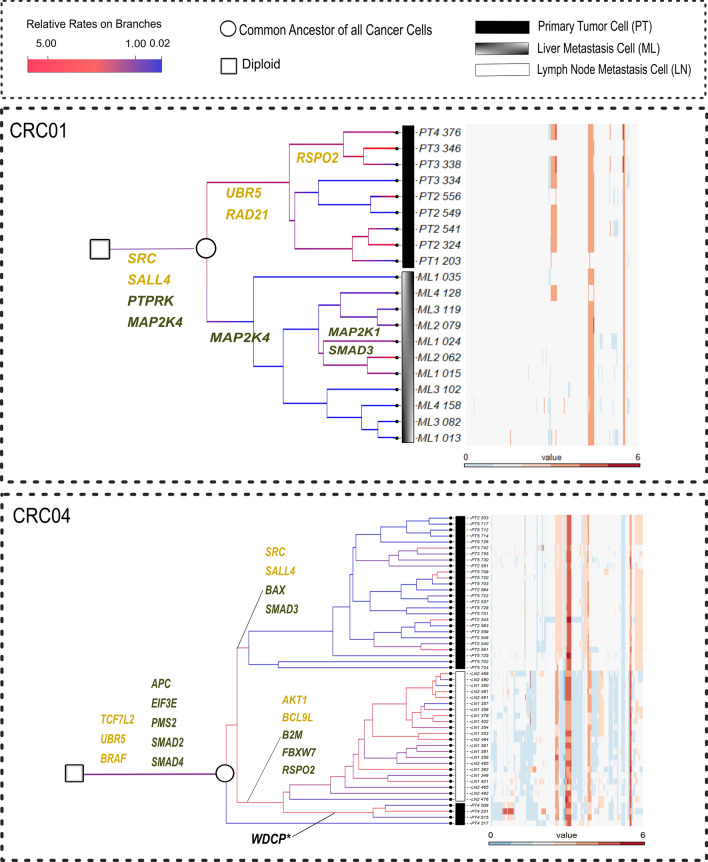
Inference results using NestedBD on data from colorectal cancer patient CRC01 and CRC04 from [34]. The heatmap shows the copy number profiles of the sampled cells. Colors of the branches indicate the estimated branch-specific relative rates from fast (red) to slow (blue). Branches defining the major cell clades are annotated with colorectal-cancer-related oncogenes (yellow) and tumor suppressor genes, or TSGs (green), impacted by CNAs. Note: while WDCP is neither an oncogene nor a TSG, the WDCP protein has been identified as a fusion protein with ALK in colorectal cancer [67]

We applied all three methods to single-cell copy number profile data sets from two colorectal cancer patients, CRC01 and CRC04, obtained from [34]. We took a subset of each data set by randomly sampling cells taken from the primary tumor and a metastasis site after excluding the cells taken from the normal adjacent tissue. For patient CRC01, we sampled 20 cells from the primary tumor site (PT) and Liver metastasis (ML), and for patient CRC04, we sampled 50 cells from the primary tumor site (PT) and Lymph node metastasis (LN). For each data set, NestedBD was run for 80 million iterations with the first 20% samples discarded as burn-in. We summarized the posterior distribution by MCC tree with median node heights and inferred the ancestral profiles and copy number profiles using the algorithm described in “Inferring corrected copy number profile” section. Support values of the inferred MCC trees are available in Additional file 1: Figs. S8 and S9. Finally, we annotated the branches that define major cell clades with the colorectal-cancer-related genes, according to [50], that were impacted by CNAs. The NestedBD inference results are shown in Fig. 7. It is worth noticing that both of the inferred trees have a relatively long branch that separates the normal cell and the most recent common ancestor of all tumor cells. This observation supports a punctuated mode of tumor evolution  [51].

#### CRC01

We detected four colorectal-cancer-related mutations on the root branch, including *SRC*, which has been shown to play an important role in the development or progression of human colon cancer and was recently postulated to be associated with liver metastasis [52]. A group of cells then metastasized to the liver after acquiring mutation of *MAP2K4*, and later a sub-lineage further acquired mutation of *MAP2K1* and *SMAD3*, resulting in a higher mutation rate. Both *MAP2K4* and *MAP2K1* are members of the *MAPK* gene family, and the *MAPK* pathway is known to be a crucial modulator of the cancer metastasis process [53]. The cells remaining at the primary tumor site evolved with a higher mutation rate after acquiring the mutations of *UBR5* and *RAD21*. We can also observe a further mutation rate increase after a group of cells acquired mutation of *RSPO2*. Although the direct relationship between these proteins and changes in mutation rate is not studied in the literature, Li et al. [54] show that there is a correlation between elevated expression of *RSPO2* in RNA samples of Patient Derived Xenograft models with colorectal cancer. Moreover, Srivastava et al. [55], in their comprehensive review discuss that *RSPO* mutations, including copy number alterations are identified in colorectal cancer samples. Similar observations have been reported for *UBR5* [56, 57] and *RAD21* [58]. We applied MP and NJ to the same data set and found neither MP nor NJ achieved such a clear separation between the PT and ML lineages (results are shown in Additional file 1: Figs. S4 and S5).

#### CRC04

Nine colorectal-cancer-related mutations are detected on the root branch of the tree, including in *APC*, a well-recognized initiator gene in colorectal cancer [59]. After that, a group of primary tumor cells acquired six additional mutations (in genes *AKT1, BCL9L, B2M, FBXW7, MUTYH, RSPO2*). An increase in the mutation rate is also observed on this branch. *FBXW7* mutations have been associated with higher tumor mutation burden in colorectal cancer [60], and *MUTYH* mutations are associated with increased lifetime risk of colorectal cancer [61]. Part of the cells then metastasized to the lymph nodes and evolved with a relatively high mutation rate; the rest remained at the primary tumor site (PT4). The rest of the cells at the primary tumor site acquired several unique mutations (in genes *SRC, SALL4, BAX, SMAD3*), but with a slower mutation rate (PT2 PT3, PT5). The association of these mutations with colorectal cancer has been identified at the level of RNA expression changes [62–65]. However, the direct relationship between these mutations and increase/decrease in mutation rate has yet to be studied. We applied MP and NJ to the same data set (results are shown in Additional file 1: Figs. S6 and S7). We observe that while MP places the same set of primary tumor cells (PT4) under the LN lineage, the topology seems to suggest those primary tumor cells are derived from the LN lineage, which is an unlikely evolutionary scenario. NJ infers a more reasonable topology similar to that inferred by NestedBD, while the branch lengths inferred by NJ do not reflect relative evolutionary time (considering that the inferred tree should be very close to ultrametric given that the cells were sampled at a single time point), as we observed that the LN lineage is closer to the diploid cell at the root in the NJ tree. This observation is consistent with the higher relative mutation rate of the LN lineage in the tree inferred by NestedBD.

## Conclusion

In this paper, we presented NestedBD, a Bayesian method for joint inference of evolutionary trees and branch lengths from scDNAseq copy number profiles. Specifically, we proposed a novel evolutionary model that uses a continuous-time birth-death process to model copy number amplification and deletion, accounting for the fact that there could be multiple CNAs at a single bin. We assume the phylogeny also follows a birth-death branching process parameterized by a diversification rate, an extinction fraction, and branch-specific mutation rates so that it is possible to distinguish between rapid expansion and slower mutations. NestedBD also infers the distribution of birth and death rates on the tree topology, the relative time between (normal) diploid cells and the most recent common ancestor of tumor cells. A major distinguishing feature of NestedBD is that it infers a tree with branch lengths representing the relative times of the tumor phylogeny nodes. NestedBD is implemented as a BEAST 2 package to utilize efficient implementation of MCMC. We assessed the accuracy of NestedBD on simulated data, demonstrated its application to biological data sets, and compared that to the results obtained by two existing methods, namely maximum parsimony and neighbor-joining. NestedBD provides more accurate results overall.

To the best of our knowledge, NestedBD is the first method to infer a tree with branch lengths that measure relative times of evolution given single-cell copy number profiles (assuming independence among bins). While the simulated data do not assume independence among bins and biological data are very unlikely to satisfy such an assumption, the results we obtained demonstrate that utilizing the independence assumption for computational efficiency does not impact the inference quality much. Recently developed methods focus on clonal tree inference, such as CONET [26] and SCICoNE [25], infer an evolutionary tree with nodes defined by CNA events jointly with breakpoints. Methods that aim to build a full binary tree, such as those reported in [29, 33, 66], infer a phylogenetic tree and reconstruct the ancestral copy number events to provide an estimate on the number of CNA events. These methods, however, do not provide information on the times of nodes and relative mutation rates per unit time per branch as NestedBD does. The ability to infer mutation rates allows NestedBD to provide insights into potential factors affecting mutagenesis, such as the hypothesis that a specific gene mutation could increase the overall mutation rate during cancer evolution. Inferred relative mutation rates could provide valuable information in evolutionary analysis of cancer cells.

A direction for future research is developing an inference method that works on the raw genomic read data directly so that it simultaneously infers the copy number profiles and evolutionary history. While such method is expected to produce the most accurate results, its scalability to large data sets could prove very challenging, which would require algorithmic innovations to achieve scalability.

### Supplementary Information

**Additional file 1: Method S1.** Settings used for maximum parsimony and neighbor joining. **Method S2.** Tree Moves. **Fig. S1.** Error in estimated copy number profiles. The Hamming distances between the true copy number profile and copy number profile estimated by Ginkgo [45] of each cell are computed and summarized across all simulated data sets. **Fig. S2.** Copy number profile simulated under different parameters. **Fig. S3.** The mutation tree inferred by SCICoNE [25] from one of the simulated data set We ran SCICoNE without performing clustering on the cells as a preprocessing step in order to acquire the mutation tree at a single-cell level and make it comparable to the results of NestedBD. However, the result shows that SCICoNE assigns all the CNA events to only one node which suggests all cells in this data set share the same copy number profile. **Fig. S4.** Inference results using MP on data from colorectal cancer patient CRC01 from [34]. The heat map shows the copy number profiles of the sampled cells and the tree is inferred by MP. At the leaves of the trees, solid rectangles correspond to primary tumor cells, and grey-gradient rectangles correspond to liver metastasis cells. **Fig. S5.** Inference results using NJ on data from colorectal cancer patient CRC01 from [34]. The heat map shows the copy number profiles of the sampled cells and the tree is inferred by NJ. At the leaves of the trees, solid rectangles correspond to primary tumor cells, and grey-gradient rectangles correspond to liver metastasis cells. **Fig.S6.** Inference results using MP on data from colorectal cancer patient CRC04 from [34]. The heat map shows the copy number profiles of the sampled cells and the tree is inferred by MP. At the leaves of the trees, solid rectangles correspond to primary tumor cells, and open rectangles correspond to lymph node metastasis cells. **Fig. S7.** Inference results using NJ on data from colorectal cancer patient CRC04 from [34]. The heat map shows the copy number profiles of the sampled cells and the tree is by NJ. At the leaves of the trees, solid rectangles correspond to primary tumor cells, and open rectangles correspond to lymph node metastasis cells. **Fig. S8.** Inferred tree using NestedBD from colorectal cancer patient CRC01 from [34], annotated with support value on each clade. (Left) phylogenetic tree inferred by the NestedBD method for the colorectal cancer patient CRC01. Each clade in the tree is annotated with its respective support value, representing the confidence level for the inferred branching patterns. (Right) histogram summarizing the distribution of these support values for all clades within the inferred tree. This figure helps assess the robustness of each branch’s inference and provides an overall confidence distribution for the tree’s topology. **Fig. S9.** Inferred tree using NestedBD from colorectal cancer patient CRC04 from [34], annotated with support value on each clade. (Left) phylogenetic tree inferred by the NestedBD method for the colorectal cancer patient CRC04. Each clade in the tree is annotated with its respective support value, representing the confidence level for the inferred branching patterns. (Right) histogram summarizing the distribution of these support values for all clades within the inferred tree. This figure helps assess the robustness of each branch’s inference and provides an overall confidence distribution for the tree’s topology.

## Data Availability

NestedBD is available at https://github.com/Androstane/NestedBD. The simulator used in this study is available at https://github.com/Androstane/SingleCellCNAsimulator. The biological data used in this study are available from European Genome-phenome Archive (EGA) under the accession number EGAS00001003242.Due to controlled access protocols established by external collaborators, our research group does not have the authority to make this data freely accessible. Additional information can be provided upon request where permissible.
