# Supplementary material for: NestedBD: Bayesian inference of phylogenetic trees from single-cell copy number profiles under a birth-death model
Source: Algorithms Mol Biol. 2024 Apr 29;19:18. doi: 10.1186/s13015-024-00264-4 (PMC11059640; doi:10.1186/s13015-024-00264-4)
Supplement: Supplementary file 1 — Additional file 1: Method S1. Settings used for maximum parsimony and neighbor joining. Method S2. Tree Moves. Fig. S1. Error in estimated copy number profiles. The Hamming distances between the true copy number profile and copy number profile estimated by Ginkgo [45] of each cell are computed and summarized across all simulated data sets. Fig. S2. Copy number profile simulated under different parameters. Fig. S3. The mutation tree inferred by SCICoNE [25] from one of the simulated data set We ran SCICoNE without performing clustering on the cells as a preprocessing step in order to acquire the mutation tree at a single-cell level and make it comparable to the results of NestedBD. However, the result shows that SCICoNE assigns all the CNA events to only one node which suggests all cells in this data set share the same copy number profile. Fig. S4. Inference results using MP on data from colorectal cancer patient CRC01 from [34]. The heat map shows the copy number profiles of the sampled cells and the tree is inferred by MP. At the leaves of the trees, solid rectangles correspond to primary tumor cells, and grey-gradient rectangles correspond to liver metastasis cells. Fig. S5. Inference results using NJ on data from colorectal cancer patient CRC01 from [34]. The heat map shows the copy number profiles of the sampled cells and the tree is inferred by NJ. At the leaves of the trees, solid rectangles correspond to primary tumor cells, and grey-gradient rectangles correspond to liver metastasis cells. Fig.S6. Inference results using MP on data from colorectal cancer patient CRC04 from [34]. The heat map shows the copy number profiles of the sampled cells and the tree is inferred by MP. At the leaves of the trees, solid rectangles correspond to primary tumor cells, and open rectangles correspond to lymph node metastasis cells. Fig. S7. Inference results using NJ on data from colorectal cancer patient CRC04 from [34]. The heat map shows the copy number profiles of the sam [file 13015_2024_264_MOESM1_ESM.pdf]

Additional Methods and Figures for

NestedBD: Bayesian Inference of Phylogenetic Trees From Single-Cell Copy Number Profile Data Under a Birth-Death Model

[1]Yushu Liu, [1]Mohammadamin Edrisi, [1]Zhi Yan, [2]Huw A. Ogilvie, [1,*]Luay Nakhleh

[1]Department of Computer Science, Rice University, Houston, Texas, USA
[2]Department of Genetics, University of Texas MD Anderson Cancer Center, Houston TX 77030
yushu.liu@rice.edu, edrisi@rice.edu, zhi.yan@rice.edu, HAOgilvie@mdanderson.org, nakhleh@rice.edu

1 Additional Methods

Method S 1. Settings used for maximum parsimony and neighbor joining

All characters are set to *ord* and diploid is set to be the outgroup. For evaluation of simulated data sets, both MP and NJ are run using [5] with bootstrap option. We set:
MP: 100 replicates are sampled from each simulated dataset. Type of search is set to *Full heuristic*. Reconnection limit is set to 8.
NJ: 100 replicates are sampled from each simulated data set. Type of search is set to *Neighbor-joining/UPGMA*. Algorithm is then set to *Neighbor-joining* with *total character difference* as the distance metric.

And for the biological data set, we set:
MP: Analysis criterion to *parsimony*. We then computed the maximum parsimony using *heuristic search* with *Maxtrees* to 100 and choose to retain only 1 tree.
NJ: Analysis criterion to *distance*. We then computed the NJ tree using *Neighbor Joining/UPGMA*. Algorithm is then set to *Neighbor-joining* with the distance option set to *total character difference*. We then selected to save branch length with the tree.

All trees are converted to rooted trees using the *outgroup* method before further evaluation.

Method S 2. Tree Moves

NestedBD uses standard tree moves implemented in BEAST 2 [2] to propose moves in tree space. The specific tree moves used in this study and their weight is summarized below:

- **TreeScaler**: scale the divergence time of tree by 0.5.The weight of this move is set to 3.0.
- **UniformOperator**: Randomly selects true internal tree node (i.e. not the root) and move node height uniformly in interval restricted by the nodes' parent and children. The weight of this move is set to 20.0.
- **SubtreeSlide**: Moves the height of an internal node along the branch. If it moves up, it can exceed the root and become a new root (but it may not exceed the diploid). If it moves down, it may need to make a choice of which branch to slide down into. The weight of this move is set to 10.0.
- **wideExchange**: Equivalent to the nearest neighbor interchange (NNI). The weight of this move is set to 10.0.
- **narrowExchange**: NNI with restriction that node height must remain consistent. The weight of this move is set to 3.0.
- **WilsonBalding**: A random subtree is moved to a new branch based on the branch-swapping move in [6]. This move is similar to SPR but acts on a rooted tree and maintains all node ages except the moved subtree. The weight of this move is set to 3.0.

2 Additional Figures

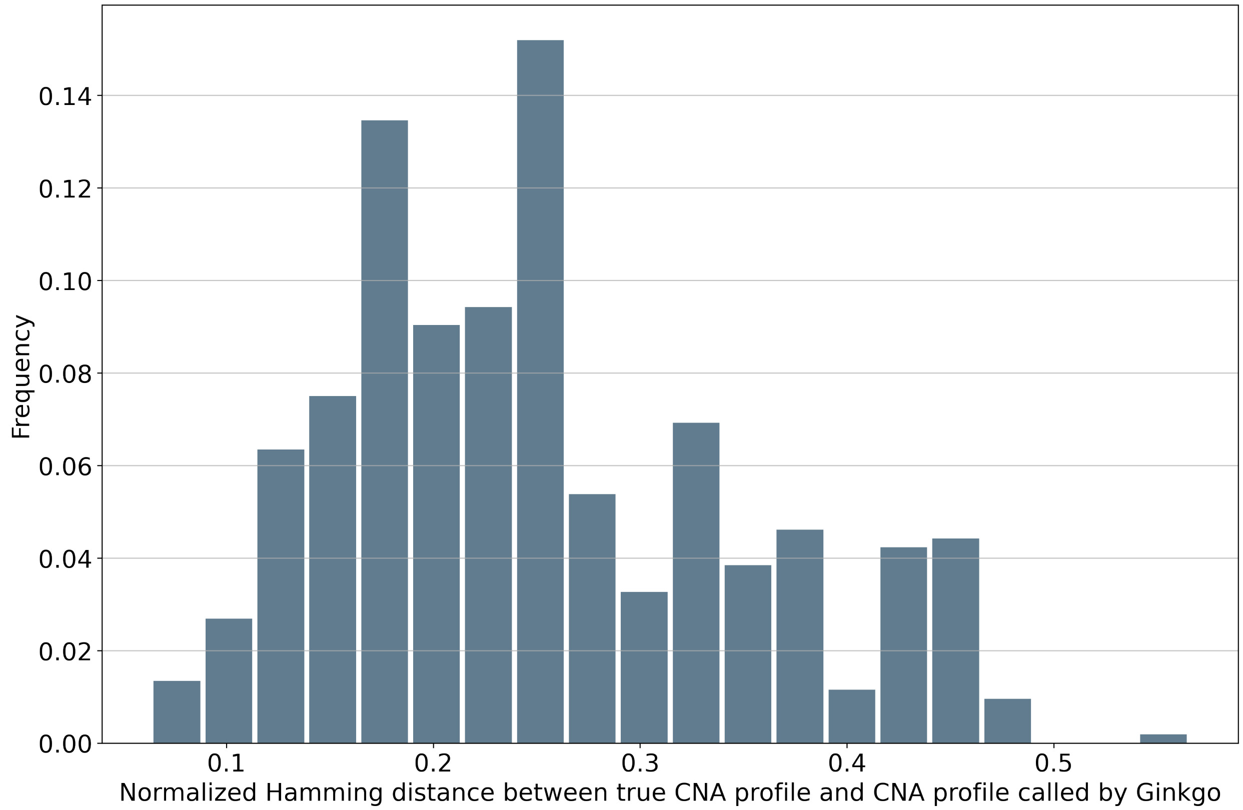

Figure S 1: **Error in estimated copy number profiles.** The Hamming distances between the true copy number profile and copy number profile estimated by Ginkgo [3] of each cell are computed and summarized across all simulated data sets.

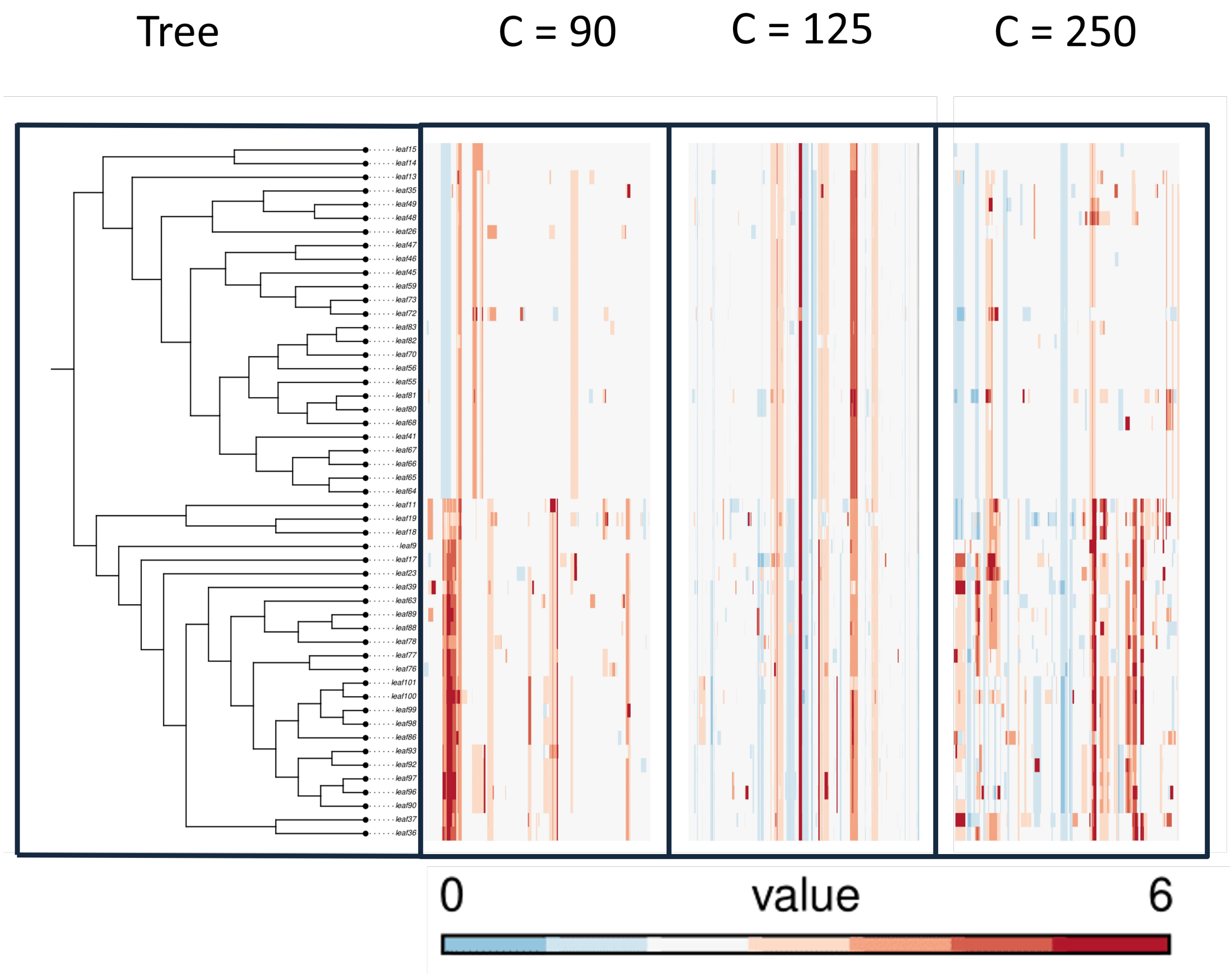

Figure S 2: **Copy number profile simulated under different parameters.**

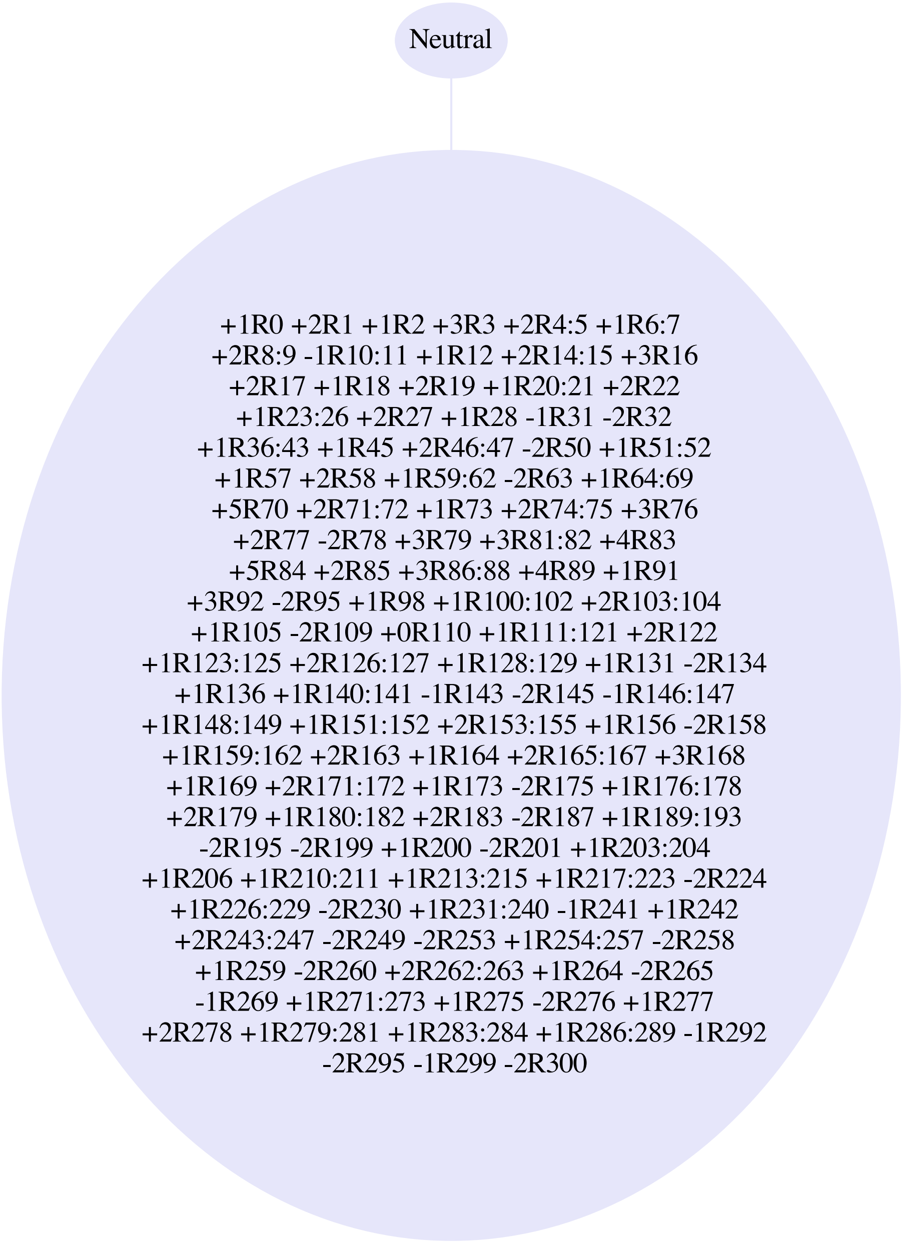

Figure S 3: **The mutation tree inferred by SCICoNE [4] from one of the simulated data set** We ran SCICoNE without performing clustering on the cells as a preprocessing step in order to acquire the mutation tree at a single-cell level and make it comparable to the results of NestedBD. However, the result shows that SCICoNE assigns all the CNA events to only one node which suggests all cells in this data set share the same copy number profile.

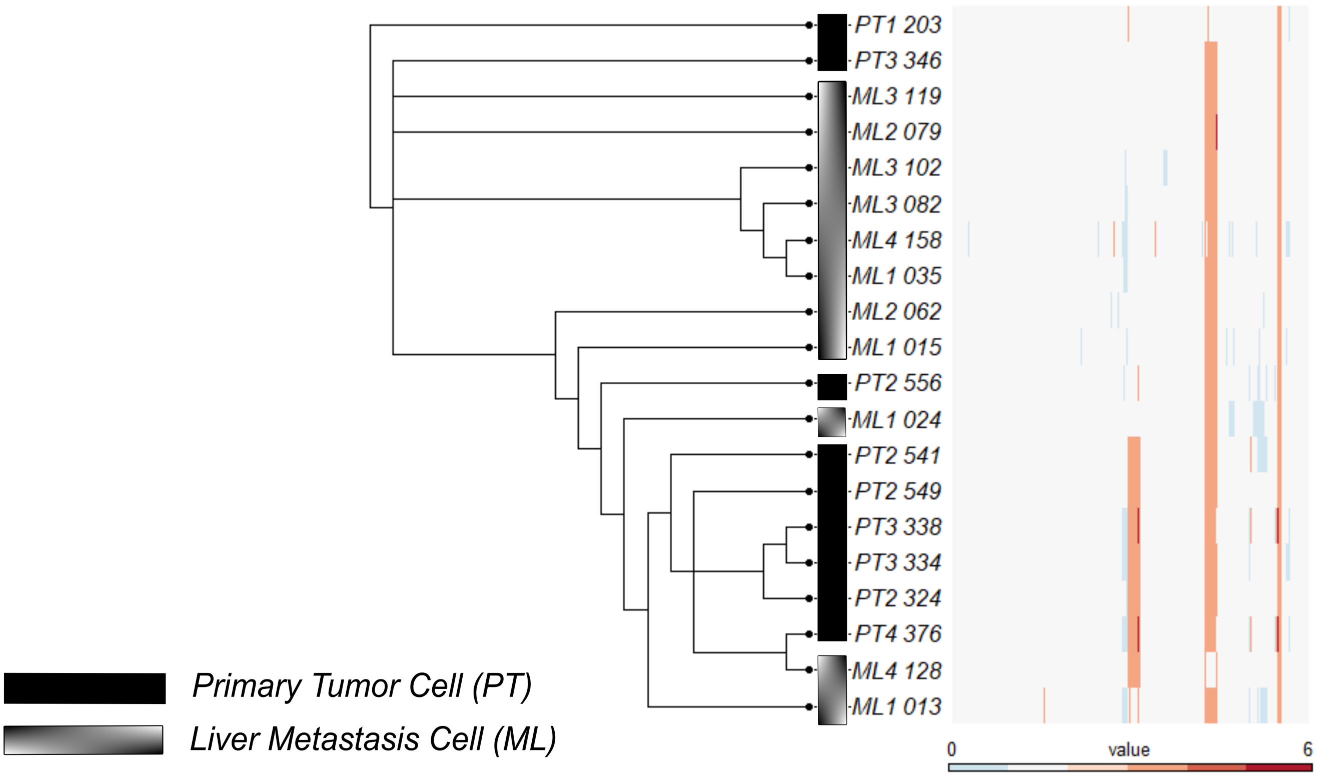

Figure S 4: **Inference results using MP on data from colorectal cancer patient CRC01 from [1].** The heat map shows the copy number profiles of the sampled cells and the tree is inferred by MP. At the leaves of the trees, solid rectangles correspond to primary tumor cells, and grey-gradient rectangles correspond to liver metastasis cells.

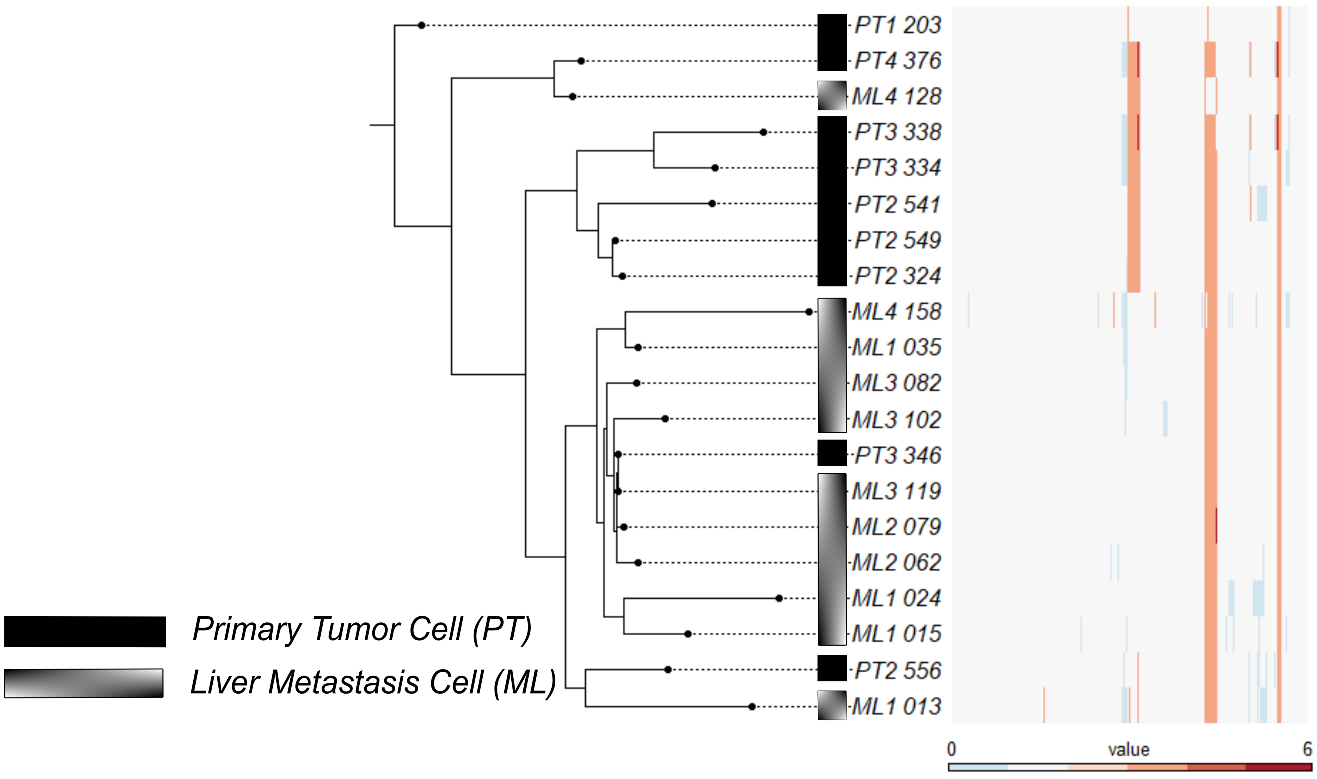

Figure S 5: **Inference results using NJ on data from colorectal cancer patient CRC01 from [1].** The heat map shows the copy number profiles of the sampled cells and the tree is inferred by NJ. At the leaves of the trees, solid rectangles correspond to primary tumor cells, and grey-gradient rectangles correspond to liver metastasis cells.

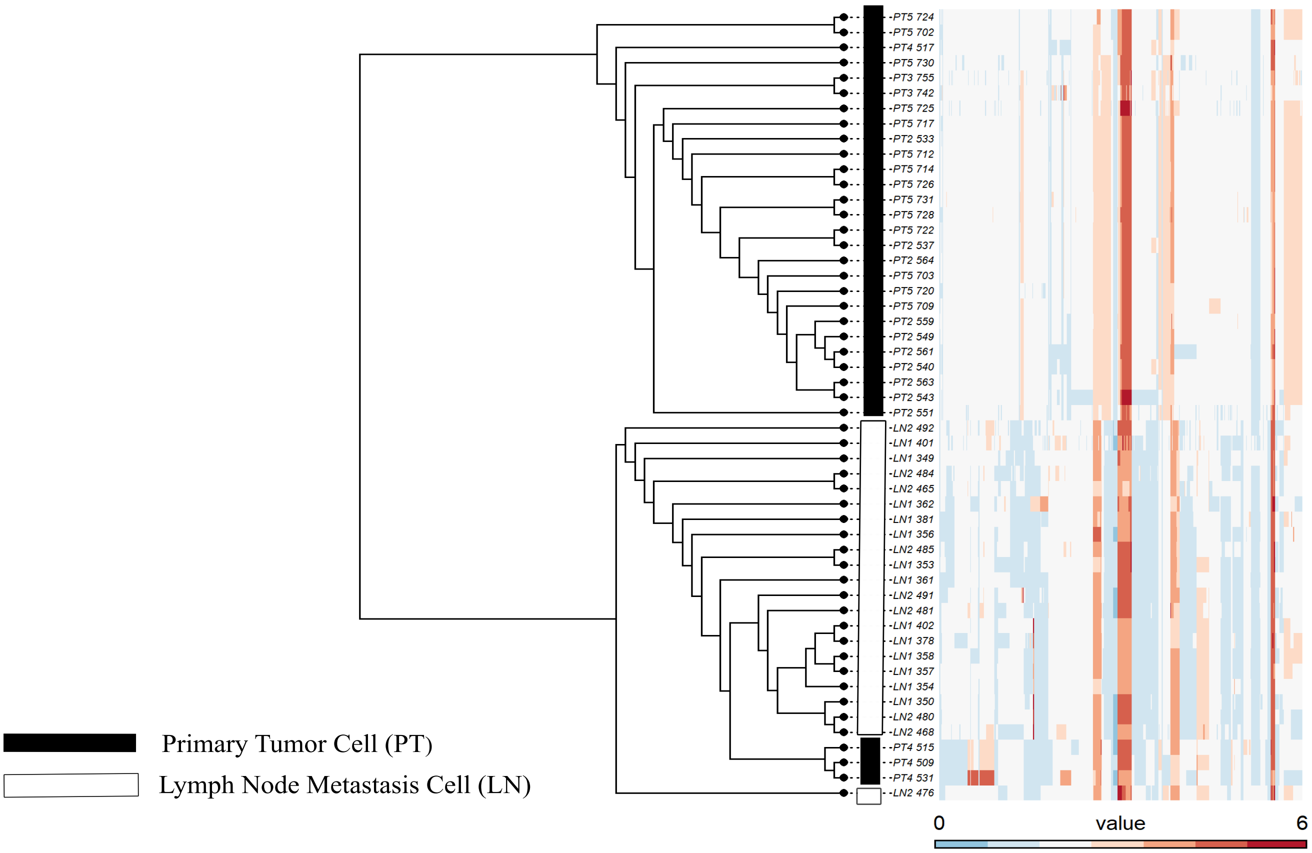

Figure S 6: **Inference results using MP on data from colorectal cancer patient CRC04 from [1].** The heat map shows the copy number profiles of the sampled cells and the tree is inferred by MP. At the leaves of the trees, solid rectangles correspond to primary tumor cells, and open rectangles correspond to lymph node metastasis cells.

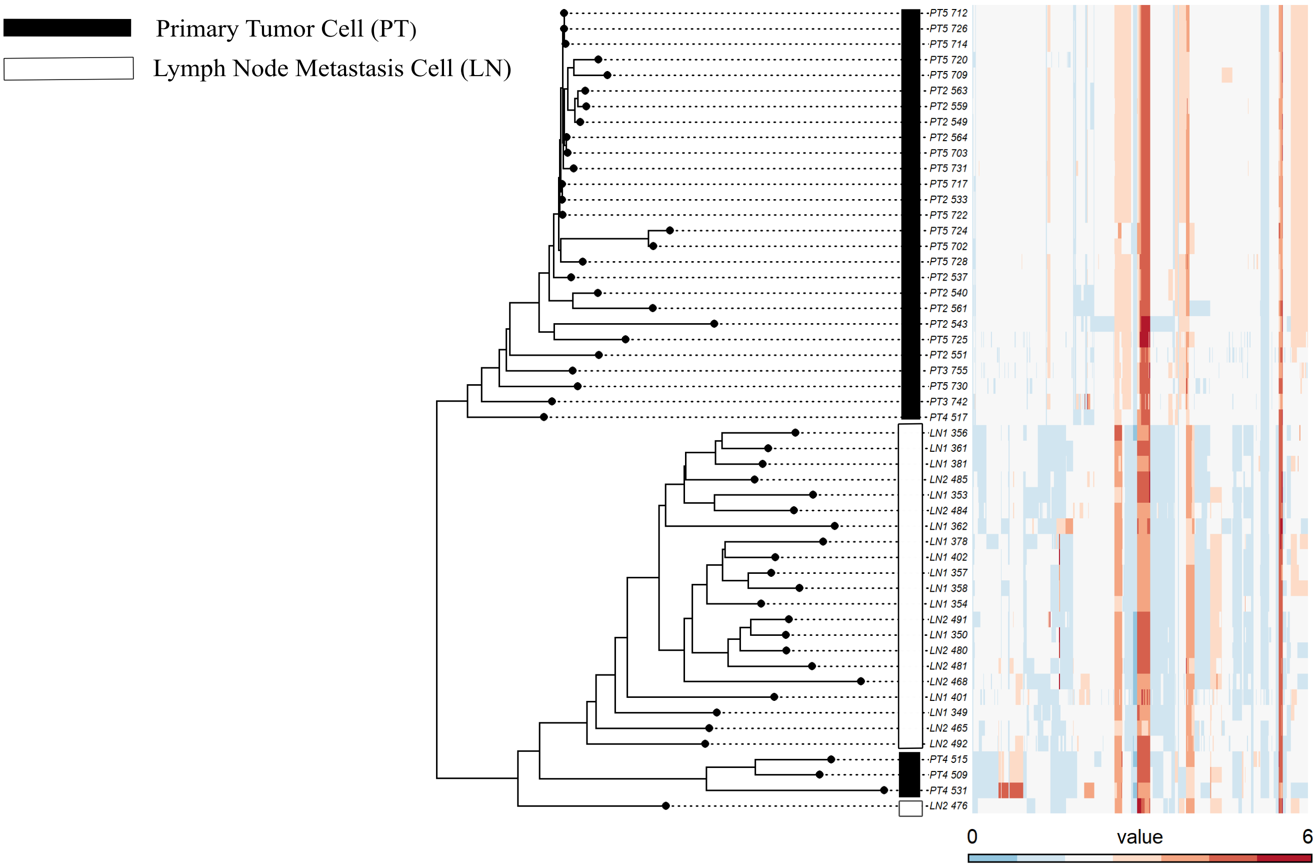

Figure S 7: **Inference results using NJ on data from colorectal cancer patient CRC04 from [1].** The heat map shows the copy number profiles of the sampled cells and the tree is by NJ. At the leaves of the trees, solid rectangles correspond to primary tumor cells, and open rectangles correspond to lymph node metastasis cells.

CRC01

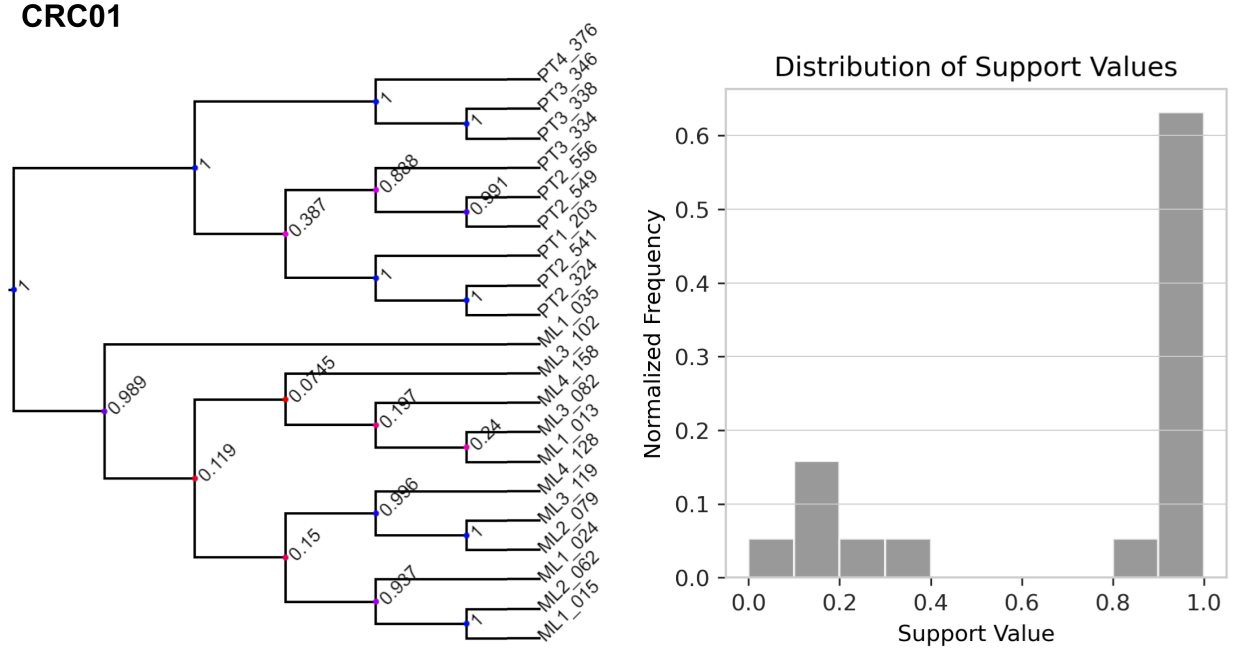

Figure S 8: **Inferred tree using NestedBD from colorectal cancer patient CRC01 from [1], annotated with support value on each clade.** (Left) phylogenetic tree inferred by the NestedBD method for the colorectal cancer patient CRC01. Each clade in the tree is annotated with its respective support value, representing the confidence level for the inferred branching patterns. (Right) histogram summarizing the distribution of these support values for all clades within the inferred tree. This figure helps assess the robustness of each branch's inference and provides an overall confidence distribution for the tree's topology.

CRC04

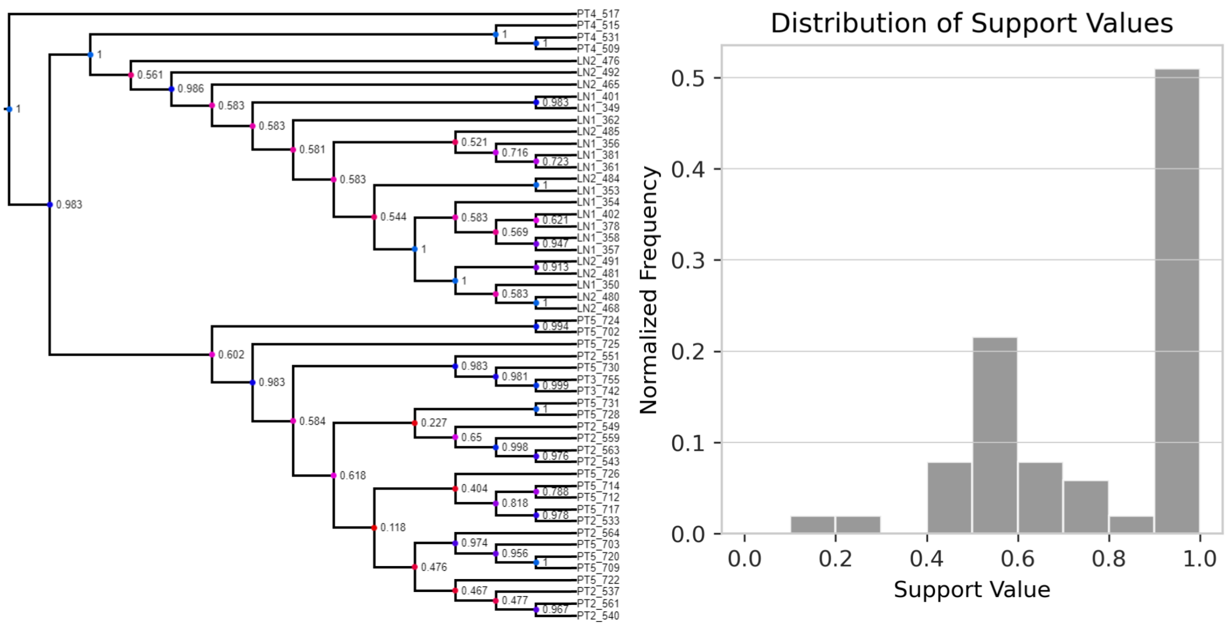

Figure S 9: **Inferred tree using NestedBD from colorectal cancer patient CRC04 from [1], annotated with support value on each clade.** (Left) phylogenetic tree inferred by the NestedBD method for the colorectal cancer patient CRC04. Each clade in the tree is annotated with its respective support value, representing the confidence level for the inferred branching patterns. (Right) histogram summarizing the distribution of these support values for all clades within the inferred tree. This figure helps assess the robustness of each branch's inference and provides an overall confidence distribution for the tree's topology.

References

[1] Shuhui Bian, et al. Single-cell multiomics sequencing and analyses of human colorectal cancer. *Science*, 362(6418):1060–1063, 2018.

[2] R. Bouckaert, et al. Beast 2.5: An advanced software platform for bayesian evolutionary analysis. *PLoS Computational Biology*, 15(4), 2019. cited By 588.

[3] Tyler Garvin, et al. Interactive analysis and assessment of single-cell copy-number variations. *Nature methods*, 12(11):1058, 2015.

[4] Jack Kuipers, et al. Single-cell copy number calling and event history reconstruction. *bioRxiv*, 2020.

[5] David L. Swofford. Paup*: Phylogenetic analysis using parsimony (and other methods) version 4.0 beta, 2001.

[6] Ian J Wilson et al. Genealogical inference from microsatellite data. *Genetics*, 150(1):499–510, 1998.
